# Like a Wave in Its Variable Shape, Breadth, and Depth: A Qualitative Interview Study of Experiences of Daytime Sleepiness in People with Parkinson's Disease

**DOI:** 10.1155/2022/9980177

**Published:** 2022-09-27

**Authors:** Arja Höglund, Peter Hagell, Ulrika Östlund, Sten Fredrikson, Christina Sandlund

**Affiliations:** ^1^Department of Clinical Neuroscience, Karolinska Institutet, Stockholm, Sweden; ^2^Department of Neurology, Karolinska University Hospital, Huddinge, Stockholm, Sweden; ^3^The PRO-CARE Group, Faculty of Health Sciences, Kristianstad University, Kristianstad, Sweden; ^4^Center for Research & Development, Uppsala University, Gävleborg, Gävle, Sweden; ^5^Faculty of Medicine and Health, School of Health Sciences, Örebro University, Örebro, Sweden; ^6^Department of Public Health and Caring Sciences, Uppsala University, Uppsala, Sweden; ^7^Department of Neurobiology, Care Sciences and Society, Karolinska Institutet, Stockholm, Sweden; ^8^Academic Primary Health Care Centre, Region Stockholm, Stockholm, Sweden

## Abstract

**Introduction:**

Daytime sleepiness is a common nonmotor symptom in Parkinson's disease (PD) which is associated with decreased quality of life and perceived health. However, experiences of daytime sleepiness in people with PD have not been explored. The aim of this qualitative study was to explore experiences of daytime sleepiness in people with PD.

**Materials and Methods:**

Five women and seven men (42–82 years) with PD for 1.5 to 21 years and excessive daytime sleepiness (i.e., a score of >10 on the Epworth Sleepiness Scale) participated in the study. Data were collected through individual, semistructured, face-to-face interviews and analyzed with qualitative content analysis.

**Results:**

Three themes of the experience of daytime sleepiness were revealed: (1) *not an isolated phenomenon,* (2) *something to struggle against or accept*, and (3) *something beyond sleepiness. Conclusion*. Daytime sleepiness is a complex nonmotor symptom in PD which manifests itself in several ways. Some experiences are similar, for instance, the attribution of daytime sleepiness to PD and its medical treatment. Differences depend on how sleepiness manifests itself, affects the person, and impacts daily life, as well as whether it causes feelings of embarrassment. Some participants needed to struggle against daytime sleepiness most of the time, and others had found a way to handle it, for example, with physical activity. However, sleepiness may also be used to benefit the person, for example, if they allow themselves to take a power nap to regain energy. The health care professionals can easily underestimate or misinterpret the prevalence and burden of daytime sleepiness because people with PD may describe daytime sleepiness as tiredness, drowsiness, or feeling exhausted, not as sleepiness.

## 1. Introduction

Parkinson's disease (PD) is an incurable neurodegenerative disorder with motor and nonmotor symptoms [1, 2]. Among the most disabling nonmotor symptoms are disturbed sleep and reduced vigilance [1]. The most common sleep-related symptoms are insomnia and poor sleep quality, and about 75% of people with PD experience such symptoms [3]. Studies suggest that sleep disturbances in people with PD are caused by disease-mediated effects on brain wakefulness [4].

Excessive daytime sleepiness (EDS) is another common symptom affecting approximately 55% of people with PD [3]. EDS is associated with sleep disorders such as insomnia, REM sleep behavior disorder, restless legs syndrome, and periodic limb movements [5]. However, there are conflicting results concerning the connection between quality of nighttime sleep and EDS [4]. Some research indicates that the pharmacological treatment of PD may contribute to EDS and sleep disturbance [4], while other studies suggest that EDS in PD is a separate, disabling nonmotor symptom [6, 7].

The American Academy of Sleep Medicine [8] defines EDS as “the inability to stay awake and alert during the major waking episodes of the day, resulting in periods of irrepressible need for sleep or unintended lapses into drowsiness or sleep. Sleepiness may vary in severity and is more likely to occur in sedentary, boring, and monotonous situations that require little active participation.” EDS is commonly identified and quantified with self-reported rating scales, for instance, the Epworth Sleepiness Scale (ESS) [9]. The ESS is an eight-item rating scale that assesses the propensity to doze off or fall asleep during various daily activities [9, 10]. Scores range from 0 to 24 (higher scores indicate more severe daytime sleepiness), and scores of >10 suggest abnormally high levels of daytime sleepiness [11].

EDS can be present prior to the diagnosis of PD [3], which suggests that it is related to the disease itself. However, the results of longitudinal studies on the progression of EDS in people with PD have been inconsistent. For example, some studies show rapid worsening of daytime sleepiness in the early stage of the disease [12, 13]. In contrast, a 10-year follow-up of people with PD found that daytime sleepiness was generally stable over time [14].

Previous studies of people with PD show that EDS can negatively impact perceived health [15]. Typically, studies investigating the prevalence and severity of EDS in people with PD have used a medical definition [9] of EDS [12, 13, 16, 17]. One qualitative study explored the burden of EDS in people with obstructive sleep apnea and found that EDS could affect health-related quality of life and daily functioning [18].

Despite the extensive research and numerous publications on the prevalence, severity, and impact of EDS, there is still a lack of knowledge about the subjective experience in people with PD, as well as what it means to live with daytime sleepiness. For example, it is not known whether people with PD and physicians are discussing the same phenomenon when they talk about sleepiness during daytime. When physicians ask about EDS, it is possible that people with PD imagine that their sleepiness must be extremely severe to fit the definition. There is also a lack of knowledge about how people with PD describe their sleepiness. Do they talk about sleepiness, tiredness, weakness, or something else? Scales such as ESS measure the propensity to fall asleep during daytime during the past month [9, 10] but not the burden of sleepiness. Maybe the burden of sleepiness is more difficult to accept and handle in daily life and has more consequences than the propensity for EDS. Do people with PD have strategies to cope with or handle this kind of sleepiness? Do they know that daytime sleepiness is a nonmotor symptom in PD, or do they think it is a natural part of aging? All these gaps in knowledge can lead to health care professionals underestimating or ignoring this nonmotor symptom in PD. More knowledge is therefore needed to understand the impact of daytime sleepiness in PD.

To the best of our knowledge, there are no qualitative studies about how people with PD experience EDS or daytime sleepiness. The aim of this study was therefore to explore the overall experiences of daytime sleepiness in people with PD.

## 2. Materials and Methods

### 2.1. Design

In this qualitative study, semistructured individual interviews were conducted with participants diagnosed with PD who had EDS according to ESS (>10 points). Data were analyzed with qualitative content analysis using an inductive approach. The interviews took place between June and October 2016 (*n* = 12). The study followed the Consolidated Criteria for Reporting Qualitative Research (COREQ) [19].

### 2.2. Sample and Recruitment

This study is a part of a larger prospective study that aimed to investigate the relationship between daytime sleepiness and nonmotor and motor fluctuations in people with PD [20]. Participants in the prospective study were recruited from a hospital outpatient clinic in Stockholm, Sweden, that specializes in PD and other neurological movement disorders. To be eligible, potential participants had to have a clinical diagnosis of PD verified by DaTSCAN and meet the criteria for EDS (>10 points on the ESS). Potential participants were excluded from the study if they had a diagnosis of severe cognitive impairment or dementia or severe untreated depression or were unable to understand Swedish.

Of the 53 participants in the original prospective study, 22 scored >10 on the ESS, which indicates excessive levels of daytime sleepiness [11]. A purposive sample of 12 participants with an ESS score of >10 (five women and seven men) of differing ages, PD duration, and PD severity were invited to participate in the current face-to-face interview study, and all accepted the invitation (Table 1).

### 2.3. Ethical Considerations

All participants provided their written informed consent before inclusion in the original prospective study [20]. The informed consent form included the information that they might be asked to take part in a qualitative interview study about experiences of daytime sleepiness. The study was conducted in accordance with the Declaration of Helsinki and was approved by the ethical review board at Karolinska Institutet, Sweden (Dnr. 2011/1866-31/4 and 2015/761-32).

### 2.4. Data Collection

#### 2.4.1. Process and Tools

At an outpatient hospital visit, participants in the prospective study completed questionnaires on daytime sleepiness [9], sleep quality [21], fatigue [22], and symptoms of anxiety and depression [23]. In all questionnaires, except the one that assesses fatigue, higher scores indicate more pronounced symptoms. Motor symptoms were assessed with the Unified Parkinson's Disease Rating Scale, motor scale (part III) [24]. Disease severity was classified in accordance with Hoehn and Yahr staging [25].

A semistructured interview guide, developed by AH and PH, was used to guide the interviews to get an overall picture of daytime sleepiness. The questions included the following: (1) Can you describe your experience of daytime sleepiness? (2) Can you tell me how daytime sleepiness affects your daily life? (3) Can you tell me if daytime sleepiness affects your Parkinson's symptoms? (4) Can you tell me how you cope with daytime sleepiness? (5) Can you tell me what words you use to describe this sleepiness? Probing questions (e.g., “Can you tell me more?”) were used to follow up and deepen responses. If the participant found it difficult to describe his or her experience of daytime sleepiness, the interviewer asked the participant to describe a situation when he or she felt sleepy during the daytime.

At the start of the qualitative study, two pilot interviews were conducted to test the interview guide. No modifications were needed, and the pilot interviews were therefore included in the study. Eleven interviews took place at the outpatient clinic and one in the participant's home, in keeping with the participants' preferences. Each participant was interviewed once. The interviews at the clinic were conducted in a separate room during office hours. Only the participant and interviewer (AH) were present during the interviews. AH had provided care for three of the participants but had not previously met the others. Before the start of each interview, AH repeated the aim of the study and confirmed the participant's willingness to take part in it.

In this study, we simplified the AASM definition in deference to the study population who may have had difficulties keeping a long definition in mind. Daytime sleepiness was thus defined as a “subjective experience of sleepiness in daytime and a tendency to fall asleep or nod off during the daytime without previously planning to go to sleep” to also capture the episodes of sudden onset of sleep. To minimize the risk of confusion about EDS and daytime sleepiness, which sometimes are used synonymously, we chose to speak about daytime sleepiness with the participants instead of EDS. Before the start of the interview, “tiredness” was explained as a lack of recovery and “sleepiness” as a need for sleep to feel recovered. This was done to explain the differences between these phenomena. These explanations were available in writing during the interview.

The interviews lasted between 10 and 35 minutes and were audio-recorded and transcribed verbatim. To limit inconvenience to participants, the transcripts were not returned to them for comment, but all were invited to contact AH after the interview if they wanted to add, change, or clarify something.

## 3. Data Analysis

Qualitative content analysis was used to analyze the data. This kind of analysis made it possible for the research group to describe the content of concrete descriptions while remaining close to the text [26]. It also enabled interpretation of latent meaning at a higher level of abstraction. The researchers thus worked simultaneously close to or distant from the text, providing more concrete (close) descriptions and more abstract (distant) interpretations [26]. An inductive approach was chosen because little is known about how people with PD experience daytime sleepiness. Before the analysis started, AH and UÖ reflected on the content of the transcripts to facilitate the choice of approach. The analysis was then conducted by AH and CS using the process described by Graneheim et al. [26, 27]. First, the text was read several times to get a sense of the whole. Then the text relevant to the aim was divided into meaning units, which were converted into shorter condensed meaning units that were labeled with a code. The codes were sorted into conceptual categories on the basis of their similarities and differences and then abstracted into themes (Table 2). During the analysis, AH and CS worked close to the original text by moving back and forth between the text, meaning units, codes, categories, and themes. This was done to maintain their awareness of the context and the essence of participants' experiences of the phenomena. AH and CS collaborated closely during the analytical process to reach consensus about the substance of the content. During this process, the analysis was discussed with PH and SF until all the authors reached consensus.

AH is a registered nurse specialized in PD. She has more than 20 years of experience providing care for people with PD and has a special interest in sleep disorders. CS is a registered nurse specialized in primary health care with expertise in sleep disorders. PH is a registered nurse specialized in PD and has a special interest in outcome measurement and the experience of living with PD. UÖ is a registered nurse specialized in oncology with expertise in cancer-related fatigue. SF is a professor of neurology and has been clinically active as a neurologist for more than 35 years.

### 3.1. Methodological Considerations

In this qualitative study, credibility, dependability, and transferability were used to discuss the study's trustworthiness [27]. Interviews were generally brief, which may be seen as a weakness. However, people with PD can have difficulty concentrating because the disease negatively impacts their cognitive functions. They may therefore prefer brief and focused interviews. Clear and focused communication between an interviewer and participants can strengthen the informational power of a study. Thus, fewer participants may be needed in focused than in unfocused dialogs [28]. Other aspects of the study also illustrate its information power. For example, open-ended questions were asked to a purposefully selected group of people with PD who experienced daytime sleepiness but had varied clinical and sociodemographic characteristics [29]. The interviews brought to light a variety of experiences, characterized by both similarities and differences. These experiences were illustrated by citations, which potentially strengthens credibility. In addition, the interviewer (AH) was aware of her prior understanding and used an open and curious approach, adding probing questions to the prepared interview guide [29]. The authors acknowledged their prior understanding (of, e.g., PD and sleep problems) to minimize the influence of prior understanding on the analysis and the interpretations. On the other hand, it is difficult to get a deeper understanding of a phenomenon if the researchers are not familiar with the topic [30]. To increase dependability, AH and CS, who conducted the main analysis, repeatedly discussed and reflected on the findings in light of their knowledge, and all authors contributed to discussions during the analysis. When they assess transferability, readers should bear in mind that the sample consisted of patients recruited from a clinic specialized in PD that was located in an urban area of Sweden. The written interviews were not returned to the participants for correction (so-called “member checking”). Maybe this would have increased the credibility of the results of this study, but out of respect for the participants' medical condition we decided to refrain. Member checking is usually done in two phases: the first is to ask participants to review the transcripts and the second is to ask them to review the first or final data analysis. However, criticism of the latter phase is that the researchers base their interpretations on several interviews, which may lead to misunderstanding when researchers and participants bring different perspectives to the analysis [30]. The sample size (*n* = 12) may seem to be low in this study but, in qualitative studies, rich content in the interviews is more important than the number of participants. In this study, the material was judged to be rich enough to fill the knowledge gaps identified about experiences of this nonmotor symptom.

## 4. Results

The analysis revealed three themes that illuminate the experiences of daytime sleepiness in the daily lives of people with PD. They experienced daytime sleepiness as (1) *not an isolated phenomenon,* (2) *something to struggle against or accept*, and (3) *something beyond sleepiness.* These themes express the experience of daytime sleepiness as like a wave in its variable shape, breadth, and depth. People with PD experience daytime sleepiness in varied ways that depend on its impact on their personal lives. Daytime sleepiness could be a driving force, and napping could provide a refreshing new start during the day.

In the quotations below, three dots indicate a pause in speech. Three dots in square brackets mean that we have omitted one or several words, and four dots in square brackets mean that we have omitted a sentence or more. Words added to the text to clarify the speaker's meaning are also indicated by square brackets.

### 4.1. Not an Isolated Phenomenon

This theme captures participants' experiences of daytime sleepiness and how other phenomena influenced this experience. They described it as a part of something bigger rather than an isolated phenomenon. Participants experienced daytime sleepiness to be related to PD itself, as a part of the disease rather than a specific motor or nonmotor symptom. They could attribute it to medical treatment for PD and link daytime sleepiness to certain situations, both monotonous and active situations. Additionally, participants experienced that the quality of nighttime sleep could have an impact on daytime sleepiness.

Participants described the relationship between daytime sleepiness, PD, and medication in several ways. “I was never sleepy during the daytime before my PD diagnosis,” said one (Participant 4). “Medication makes me very sleepy, I can hardly stay awake,” said another (Participant 12). Participants described themselves as not more tired and sleepy than others of their own age. However, it was more difficult to them to resist sleepiness during boring or monotonous situations than it had been before their PD diagnosis.

Some situations, certain places, and monotonous activities could prompt sleepiness, which could even feel irresistible. However, similar activities might not lead to the same sleepiness if the person enjoyed them. An example was driving a motorboat (enjoyable) as opposed to driving a car (monotonous).

Daytime sleepiness fluctuated during the day. For example, they could feel alert in the morning but hardly able to wake up later or sleepy all day. Sleepiness was most obvious to them in the evening or in passive situations, such as resting after an activity, but it also occurred during more active situations, such as during meetings and when driving. Sleepiness could also be like a barrier that they had to overcome to feel more alert.

“I don't exactly go to the doctor and lie down. So, there I become alert. So it's like some kind of barrier that somehow lets go, and so I become alert, but I really want to sleep for a while” (Participant10).

They also connected their ability to handle daytime sleepiness to the quality and length of their nighttime sleep. If they had slept well at night, they could tolerate daytime sleepiness better. One person (Participant 12) said, “It [daytime sleepiness] comes, but it depends on how much I slept at night, so how prepared I am to take that sleepiness can be different.”

### 4.2. Something to Struggle against or Accept

This theme captures participants' experiences of daytime sleepiness as something to struggle against or accept or to find strategies to cope with. Some participants felt exhaustion, fatigue, and an overwhelming need to fall asleep. They might have to struggle with sleepiness several times a day. Others had found a way to accept their sleepiness, for example, by finding different kinds of activities that could push it away or by seeing benefits in sleepiness. At the same time, participants could have conflicting feelings about and varied experiences of daytime sleepiness.

One participant described overwhelming sleepiness as “Like cotton in my head. I never feel awake” (Participant 5). Sleepiness could even be paralyzing, something that took over life and was nearly impossible to fight against. Another described it as follows:

“Now I'm going to knit and lay it [knitting materials] on the sofa, but then I don't have the energy to do it after all. I just sit there [....] Then I just fall asleep” (Participant 1).

As noted, one way to gain acceptance was to figure out strategies to push away or reduce sleepiness with activity. For example, during meetings or other passive situations, it was not enough to change position. Participants needed more vigorous activity to counteract sleepiness, such as rising from a seated position or walking around.

Physical activity was a common way to handle and resist daytime sleepiness. One strategy was to plan activities or keep moving most of the day because it felt like physical activity could push away sleepiness. One person said, “I don't fall asleep while I am walking around” (Participant 11). Another said, “I have to move when I get sleepy like that, and then it feels good, then I feel like I could run around for hours doing things” (Participant 4). At the same time, some very active participants could unexpectedly fall asleep when they sat down. Another had a hard time staying active because the sleepiness was so hard to resist. It was like he had become “stuck in a sleep corridor” and had to fight to keep himself awake. It was difficult to affect sleepiness only with mental activity unless it was interesting or engaging in some way, in which case the person could feel more alert and forget about their sleepiness.

Participants could also accept the feeling but not allow it to disturb their daily life. Even when the experience of daytime sleepiness did not disturb daily life, the feeling of losing control during sleepiness was uncomfortable. Another way to talk about daytime sleepiness was to minimize it but acknowledge that it was a potential danger, for instance, while driving. Participants also expressed a contradictory experience, such as some level of acceptance coupled with a feeling of worry.

“Yeah, it actually sucks to fall asleep whenever. It's uncomfortable. But for me personally, I think it's uncomfortable, but take, I don't take it as something negative, so it's a conflict for me [...] [I] don't take what happens so seriously, but really, it's uncomfortable to disappear without wanting to” (Participant 3).

Napping could also be seen as positive, a way to feel refreshed and restart the body and brain. Participants said that they “have all the right to rest or sleep for a while” (Participant 8) and that it was fine “to prioritize a nap” (Participant 6) or “take a nap to refresh and restart my body and brain” (Participant 12).

“I love to take naps. I love to be allowed to fall asleep. So, I really look forward to it, and therefore I don't want to book up these times. Instead, I want, I rush home to manage to sit down for this afternoon nap” (Participant 6).

### 4.3. Something beyond Sleepiness

This theme captures descriptions and consequences of daytime sleepiness in participants' daily life. The phenomenon was complex to describe. None of the participants described their sleepiness as only sleepiness. Instead, they talked about a hard-to-resist mixture of tiredness, sleepiness, and fatigue. Daytime sleepiness could affect self-image. Participants could feel that their sleepiness made them less valuable in the eyes of others and repeatedly brought up losing control when they talked about sleepiness. Even mental and physical functions could be worsened by daytime sleepiness. Several said that daytime sleepiness limited their daily lives, both privately and professionally.

Some participants described their experience as tiredness rather than sleepiness. Some explained that “sleepiness” was not enough to describe the sensation. It was too mild a term. They felt sleepy, but the feeling was part of a larger tiredness, something beyond sleepiness. It was a combination of sleepiness, tiredness, and fatigue and was therefore difficult to name. One described it as drowsiness: “I'm drowsy all the time. I never [fully] wake up during the day [....] No matter how much I rest, it doesn't go away” (Participant 5). Another said: “Sleepiness can be an aspect of tiredness. So, I see tiredness as a larger concept that has different facets. So, I would describe it as being tired and sleepy – sleepy-tired. I don't know what I should say. Sleepy-tired, but I'm more, more tired than before, and this tiredness manifests itself as sleepiness” (Participant 11).

Participants' self-images could change for the worse. One said, “I can't take it... this isn't me” (Participant 1). They described feeling lazy, uninterested in others, and worried that other people noticed their sleepiness (e.g., during meetings): “I'm not aware that I'm dropping off [....] And it's disturbing for the others too, if a person sits and sleeps. It's impolite” (Participant 9). They were also worried about losing control when they felt irresistible sleepiness.

Tiredness and sleepiness could also be experienced as a physical sensation. One person described it in the following way:

“I would like to take this off... like a coat. Some days, it feels like a big bird taking me in it its powerful claws and hugging my body so hard. Hugging and not letting go. Then the day after, it can be like it lets go, and I feel much better for a while [....] Some days are just completely lost” (Participant 1).

Daytime sleepiness could affect even mental functioning. One of the participants said, “When I feel sleepy, I'm not able to solve the most rudimentary mathematical problems like plus and minus” (Participant 11). Another (Participant 12) described losing his judgment when he was in the process of falling asleep. Daytime sleepiness could lead to loss of focus and thus to feelings of embarrassment and could be described like:

“Yes, losing focus – that's at the root of all of it. The sleepiness gets worse then. I lose the thread, and it's hard to find my way back. And it can be really hard when you're sitting and discussing something. It can be so embarrassing that I prefer to refrain from talking then. You lose concentration or forget a memory or whatever it is. I don't know what it is” (Participant 11).

## 5. Discussion

To the best of our knowledge, this is the first study to explore the experiences of daytime sleepiness in people with PD. Twelve people with PD and EDS (according to the ESS) were interviewed. They experienced daytime sleepiness like a wave in its variable shape, breadth, and depth rather than as an isolated, single phenomenon. The phenomenon was something to struggle against or accept or cope with and could be mastered by different strategies for some participants. It was bigger than just feeling sleepy, something beyond sleepiness.

Participants related sleepiness to having PD and to the treatment they received for the disease. Research shows that PD and its treatment can affect the basic diurnal variation of sleep and wakefulness [4]. They did not relate their sleepiness to the progression of PD over time, which is consistent with the results of a previous longitudinal study that found that EDS did not deteriorate during disease progression [14]. However, other studies have found that EDS increased in severity during the progression of PD [12, 13, 31].

None of the participants expressed the idea that their motor or nonmotor symptoms were directly linked to sleepiness. This finding was unexpected, as several studies [14, 20, 32, 33] have found a correlation between EDS, motor symptoms, and nonmotor symptoms (e.g., depression and anxiety) in people with PD. However, such associations are not necessarily causal and have in general not been particularly strong [6, 14, 32].

According to our findings, the burden of sleepiness could vary during the day and over time. Participants could feel sleepier or even fall asleep during monotonous situations and in the evening. Those who found it easier to accept the sleepiness and used strategies to cope with it may have had a higher level of perceived resilience than those who described their sleepiness as more severe and overwhelming. Few studies have investigated the role of resilience in the experiences of people with PD. One that did found that resilience correlated with nonmotor symptoms like depression, fatigue, and anxiety, as well as with having an optimistic personality [34]. In this study, we did not explore these phenomena or their relationship with sleepiness, but future studies could investigate this topic.

Participants who described their daytime sleepiness as severe and something to struggle against most of the time also described physical symptoms and difficulty thinking during episodes of sleepiness. Their experiences of sleepiness may be related to fatigue or a combination of fatigue and daytime sleepiness. Both fatigue and daytime sleepiness are common nonmotor symptoms in PD, and although their definitions differ, the two phenomena appear to overlap [35, 36]. Daytime sleepiness is characterized by feeling sleepy and at risk of falling asleep, whereas fatigue is characterized by a lack of energy and exhaustion linked to physical and cognitive impairment [37]. If these feelings were experienced simultaneously, it could be difficult to distinguish one from the other.

PD may be a risk factor for social isolation because of symptoms such as impaired communication, including reduced facial and bodily expressions [38]. Daytime sleepiness may further limit social contacts and thus increase isolation. In this study, participants who felt that sleepiness reduced social contacts with others also worried that their family members could become isolated. Moreover, participants associated daytime sleepiness with being seen as lazy and less valuable in the eyes of others. These feelings of embarrassment may have contributed to their altered self-image, which might lead to even greater social isolation [39]. These findings are in line with those of a previous study that explored the experiences of EDS in people with obstructive sleep apnea [18].

Participants in this study found it difficult to describe their feelings of sleepiness. For example, they called their sleepiness “tiredness” because they found that “sleepiness” was too limited a term to express the feeling. They explained their sleepiness as something beyond feeling sleepy, like a combination of sleepiness, tiredness, and fatigue. This underscores the importance of previous recommendations that clinicians ascertain what people with PD mean when they say they feel “tired,” “fatigued,” “sleepy,” “groggy,” or “drowsy” [40]. Additionally, many participants had not discussed their daytime sleepiness with their health care practitioner because they did not know that it is a common symptom in PD. This finding is consistent with that of a cohort study that suggested that people with PD may connect daytime sleepiness to poor nighttime sleep and therefore may not bring it up during medical consultations [41].

### 5.1. Relevance to Clinical Practice

Although daytime sleepiness is a well-known symptom in PD, the results of this study illustrate that it also is a hidden problem. Daytime sleepiness is not an isolated nonmotor symptom; it is more complex and has several dimensions related to PD and its medical treatment. It has consequences for daily life and can be difficult to resist and accept. There are several reasons why daytime sleepiness is still a hidden problem in people with PD. One is that people with PD do not always identify their sleepiness as a symptom, rather than a part of the process of aging. Another is that they can use unspecific and mild-sounding terms such as “tiredness” and “drowsiness” to describe what they are experiencing. On the other hand, when clinicians ask about EDS, it is possible that they use the medical definition of “severe daytime sleepiness” and that, as a result, people with PD imagine that their sleepiness must be extreme to fit the definition. Thus, there is a risk that clinicians and patients may misunderstand each other, resulting in an underestimation of the presence and burden of daytime sleepiness in people with PD. It is important for clinicians to give people with PD the opportunity to describe their experiences of daytime sleepiness by asking in multiple ways about what the patient means when he or she mentions tiredness and/or sleepiness and whether these experiences have consequences in the patient's daily life. It may also be important to invite family members to the discussion to obtain a broader picture of the daytime sleepiness and its impact on everyday life [42]. Instruments such as the ESS can be used to detect daytime sleepiness and assess its severity, but they do not assess the burden or consequences of daytime sleepiness. They should be used as a complement to, rather than a substitute for, discussions with patients about their experiences.

The participants in this study found that physical activity could both relieve and induce sleepiness. Thus, recommendations about physical activity should be individualized, preferably in consultation with a physiotherapist. Self-management education might also help people with PD manage daytime sleepiness. Such education is available in many countries [43], including Sweden, where the National Parkinson School provides a scientifically evaluated educational program to help people with PD and their partners live and cope with the disorder [44].

PD is a very individual disease. In people with PD experience of similar symptoms, both motor and nonmotor, a difference may exist between the individuals and how clinicians assess these. For example, a clinician can assess a tremor as discrete, whereas the patient experiences it as severe and troublesome. A person-centered care approach can help clinicians better understand how each patient experiences his or her symptoms and their impact on daily life [45]. Such an approach could even lead to better adherence to medical treatment and opportunities to facilitate care and self-care based on the individual's needs [45]. People with PD and daytime sleepiness need to be approached individually because of the multidimensional expression of this bothersome phenomenon.

Further research is needed on the topic of daytime sleepiness in people with PD. We investigated people who scored more than 10 points on the ESS, the definition of EDS. There is no qualitative data about the burden and consequences of symptoms of daytime sleepiness on daily life in people with PD who score 10 or fewer points on this instrument. Future studies should investigate whether such people have similar experiences of daytime sleepiness. There is also a need for an instrument to measure the burden of daytime sleepiness, perhaps in the form of a virtual analog scale to measure the burden of sleepiness. Such a scale could potentially be added to the ESS.

## 6. Conclusions

Daytime sleepiness is a complex nonmotor symptom in PD which manifests itself in several ways. Some experiences are similar, for instance, the attribution of daytime sleepiness to PD and its medical treatment. Differences depend on how sleepiness manifests itself, affects the person, and impacts daily life, as well as on whether it causes feelings of embarrassment. Some participants needed to struggle against daytime sleepiness most of the time, and others had found a way to handle it, for example, with physical activity. However, sleepiness may also be used to benefit the person, for example, if they allow themselves to take a power nap to regain energy. Health care professionals can easily underestimate or misinterpret the prevalence and burden of daytime sleepiness because people with PD may describe daytime sleepiness as tiredness, drowsiness, or feeling exhausted, not as sleepiness.

## Figures and Tables

**Table 1:** Sample characteristics (*n* = 12)^a^.

Variable	Participants
Gender (female/male) (*n*)	5/7
Age (years)	65.0 (61.0–75.75; 42–82)
Time since PD diagnosis (years)	6.5 (2.6–10.75; 1.5–21)
Hoehn & Yahr stage of PD inON (I–V)^b,c,d^	2.5 (I-II; I-III)
Hoehn & Yahr stage of PD inOFF (I–V)^b,d^	III (III-IV; III-IV) (*n* = 7)
ESS daytime sleepiness score (0–24)	14 (13–19; 12–23)
PSQI, sleep quality score (0–21)^d^	10.0 (9.25–13.0; 9–14)
FACIT-F, fatigue score (0–52)^e^	34.0 (26.75–37; 12–47)
HADS, depression score (0–21)^d^	9.0 (7.25–12.5; 6–15)
HADS, anxiety score (0–21)^d^	13.0 (10.25–15.0; 9–15)

^a^Data are median (q1–q3; min–max) unless otherwise noted. ^b^Range, I–V (I = mild unilateral disease; II = bilateral disease without postural impairment; III = bilateral disease with postural impairment, moderate disability; IV = severe disability, still able to walk and stand unassisted; V = confined to bed or wheelchair unless aided). ^c^As assessed during the “ON” phase. ^d^Higher scores = worse. ^e^Higher scores = better. PD, Parkinson's disease; ESS, Epworth Sleepiness Scale; PSQI, Pittsburgh Sleep Quality Index Profile; FACIT-F, Functional Assessment of Chronic Illness Therapy—Fatigue scale; HADS, Hospital Anxiety and Depression Scale.

**Table 2:** An overview of the analytical process, including examples of meaning units, codes, categories, and themes.

Meaning units	Condensed meaning units	Codes	Categories	Themes
I was never sleepy in the daytime before my PD diagnosis (Participant 4)	Never sleepy before PD	Disease	Related to the disease	Not an isolated phenomenon
[Daytime sleepiness] comes, but it depends on how much I slept at night, so how prepared I am to take that sleepiness can be different (Participant 12)	Quality of nighttime sleep is essential	Sleep quality	Related to internal factors	
But there is a difference (drive car/boat) because I drive a boat a lot. I never get tired then [....] I have never had this kind of problem when I have driven a boat (Participant 3)	The enjoyability of situation can play a role	Situational	Related to external factors	

I have to move when I get sleepy [....] I could run around for hours doing things. And that feels good (Participant 4)	Fight against sleepiness with physical activity	Activity	Coping strategies	Something to struggle against or accept
Yeah, it actually sucks to fall asleep whenever. It's uncomfortable [....] I think it's uncomfortable, [....] I don't take it as something negative, so it's a conflict for me (Participant 3)	Conflicting thoughts about daytime sleepiness	Conflict ignore	Attitude	
Take a nap to refresh and restart my body and brain (Participant 12)	Restart body and brain	Power	Coping strategies	

I can't take it […] this isn't me (Participant 1)	This isn't me	Self-image	Personality change	Something beyond sleepiness
Yes, losing focus—that's at the root of all of it. The sleepiness gets worse then. Lose the thread, and it's hard to find my way back [....] It can be so embarrassing that I prefer to refrain from talking then (Participant 11)	Losing focus is so embarrassing that I prefer to refrain from talking	Shame	Negative feelings	
I see tiredness as a larger concept that has different facets [....] as being tired and sleepy—sleepy-tired [....] and this tiredness manifests itself as sleepiness (Participant 11)	Sleepiness is a larger concept than only sleepiness	Larger concept	More than sleepiness	

## Data Availability

The protocol and qualitative analysis data used to support the findings of this study are available from the corresponding author upon request. Deidentified participant data are not available for legal and ethical reasons. The prospective study is included in a doctoral thesis about daytime sleepiness in people with PD [46].
